# Influenza A Virus Hemagglutinin and Other Pathogen Glycoprotein Interactions with NK Cell Natural Cytotoxicity Receptors NKp46, NKp44, and NKp30

**DOI:** 10.3390/v13020156

**Published:** 2021-01-21

**Authors:** Jasmina M. Luczo, Sydney L. Ronzulli, Stephen M. Tompkins

**Affiliations:** 1 Center for Vaccines and Immunology, University of Georgia, Athens, GA 30602, USA; jasmina.luczo@csiro.au (J.M.L.); slronzulli@uga.edu (S.L.R.); 2 Emory-UGA Centers of Excellence for Influenza Research and Surveillance (CEIRS), Athens, GA 30602, USA; 3 Department of Infectious Diseases, University of Georgia, Athens, GA 30602, USA

**Keywords:** influenza, hemagglutinin, natural killer cells, natural cytotoxicity receptors, NKp46, NKp44, NKp30

## Abstract

Natural killer (NK) cells are part of the innate immunity repertoire, and function in the recognition and destruction of tumorigenic and pathogen-infected cells. Engagement of NK cell activating receptors can lead to functional activation of NK cells, resulting in lysis of target cells. NK cell activating receptors specific for non-major histocompatibility complex ligands are NKp46, NKp44, NKp30, NKG2D, and CD16 (also known as FcγRIII). The natural cytotoxicity receptors (NCRs), NKp46, NKp44, and NKp30, have been implicated in functional activation of NK cells following influenza virus infection via binding with influenza virus hemagglutinin (HA). In this review we describe NK cell and influenza A virus biology, and the interactions of influenza A virus HA and other pathogen lectins with NK cell natural cytotoxicity receptors (NCRs). We review concepts which intersect viral immunology, traditional virology and glycobiology to provide insights into the interactions between influenza virus HA and the NCRs. Furthermore, we provide expert opinion on future directions that would provide insights into currently unanswered questions.

## 1. Introduction

Natural killer (NK) cells are considered an ancient cytotoxic immune effector cell involved in the innate immune response. It was about 45 years ago that these cells were characterized for their non-specific cytotoxic abilities [1,2,3]. NK cells develop mainly in the bone marrow from a common lymphoid progenitor [4]. They are large, granular lymphocytes that comprise approximately 15% of lymphocytes in the human circulatory system [5,6,7]. Unlike, B and T lymphocytes that express somatically rearranged receptors that specifically target one antigen antigen [8,9,10], NK cells express germline encoded activating and inhibitory receptors that do not undergo receptor rearrangement [11,12], and function to recognize various ligands expressed on target cells [13,14,15].

NK cells are the frontline of defense in an immune response against many pathogens, including intracellular and extracellular bacteria, protozoa, fungi, and viruses (reviewed in [16,17,18,19]). The critical role for NK cells against viral pathogens is highlighted by a case report of a patient lacking NK cells whom was highly susceptible to herpesvirus infection [20]. Mice lacking a key NK cell activating receptor, natural cytotoxicity receptor (NCR) 1, are highly susceptible to influenza infection [21,22,23]. NK cells can kill transformed or pathogen-infected cells through the secretion of cytokines and chemokines, or by directly lysing the target upon recognition via numerous activating receptors. A delicate balance of signals relayed through activating and inhibitory receptors on NK cells is important to determine the outcome of the interaction; whether target cells will be killed via the release of cytotoxic granules (containing perforin and granzymes), via death receptors that cause direct lysis, or spared [24,25,26].

NK cell activating and inhibitory receptors are grouped into three major superfamilies: (1) the C-type lectin superfamily, which primarily recognizes non-classical human leukocyte antigen (HLA)-E on transformed or foreign cells, (2) the killer-cell immunoglobulin-like receptor (KIR) superfamily, which recognizes classical HLA-A through HLA-C on transformed or foreign cells, and (3) the NCRs that recognize target cells in a HLA-independent manner [27]. The NCR family of activating receptors include NKp46 (also known as NCR1, cluster of differentiation (CD) 335), NKp44 (NCR2, CD336), and NKp30 (NCR3, CD337), and recognize various, sometimes unknown, ligands on transformed, foreign, or pathogen-infected cells.

Viral glycoproteins from multiple virus families have been reported to interact with NCRs, including members of *Orthomyxoviridae* (influenza A virus (IAV) [21,22,23,28,29,30,31,32,33,34,35,36,37] and influenza B virus (IBV) [29]), *Paramyxoviridae* (Sendai virus (SeV) [28,29,38,39], human parainfluenza virus 3 (HPIV3) [37], and Newcastle disease virus (NDV) [40]), *Pneumoviridae* (human metapneumovirus (HMPV) [41]), *Herpesviridae* (human cytomegalovirus (HCMV) [42], herpes simplex virus 1 (HSV1) [43] and Kaposi’s sarcoma-associated herpesvirus (KSHV) [44]), *Poxviridae* (*Vaccinia virus* (VACV) [45,46], *Cowpox virus* [45], *Camelpox virus* [45] and *Ectromelia virus* (ECTV) [46]), and the *Flaviviriviridae* family of viruses (Dengue virus (DENV) and West Nile virus (WNV) ) [47]) (Table 1). Moreover, interactions between NCRs and bacterial and parasitic pathogens have been described for *Mycobacterium* [48,49,50], *Plasmodium falciparum* [51], *Enterococcus faecium* [50], and *Fusobacterium nucleatum* [33] (Table 1). Of these pathogens, the interactions between the IAV hemagglutinin (HA) glycoprotein and human NCRs has been the most extensively studied.

The mechanisms underlying the recognition of IAV by NK cells is under extensive investigation. In this review, we describe NK cell functional biology, NK cell NCR glycobiology, the role of NK cells in influenza disease outcomes, influenza virus biology with a particular focus on HA structure and function, and the interactions of influenza virus HA and NK cell NCRs. We summarize current knowledge regarding the carbohydrate-mediated interactions of influenza virus HA with the NK cell NCRs, currently unanswered questions, and provide discussion as to future directions.

## 2. NK Cell Functional Biology

NK cells, regardless of phenotype, survey and eliminate aberrant cells that fail to express major histocompatibility complex (MHC) class I ligands, a process known as the “missing self” hypothesis [4,11,63]. When NK cells were first described in the 1970s [1,2], it was thought that these cells were not MHC-restricted [11]. However, Ljunggren and Karre reported that NK cells were activated or inhibited depending on MHC I expression on tumor cells [64]. Furthermore, NK cells were demonstrated to have specificity to MHC class I ligands [65,66]. Following this discovery, it was thought that NK cells are inhibited or activated through recognition of MHC class I, a misconception. MHC class I inhibitory receptors may only serve to dampen the response, rather than completely inhibit NK cell function [11].

Many pathogens, particularly viruses, downregulate MHC class I on the surface of infected cells, rendering them targets for NK cell recognition and lysis (reviewed in [67]). While aspects of the activation of NK cells that lead to lysis of target cells are relatively well described, the processes by which NK cells acquire the ability to act on cells that fail to express self MHC are not completely understood. Recent findings suggest that although NK cells do not undergo receptor rearrangement and selection (in contrast to T and B cells), licensing via inhibitory receptors and MHC Class I molecules is required for development into cytotoxic NK cells (reviewed in [68]).

**Figure 1:**
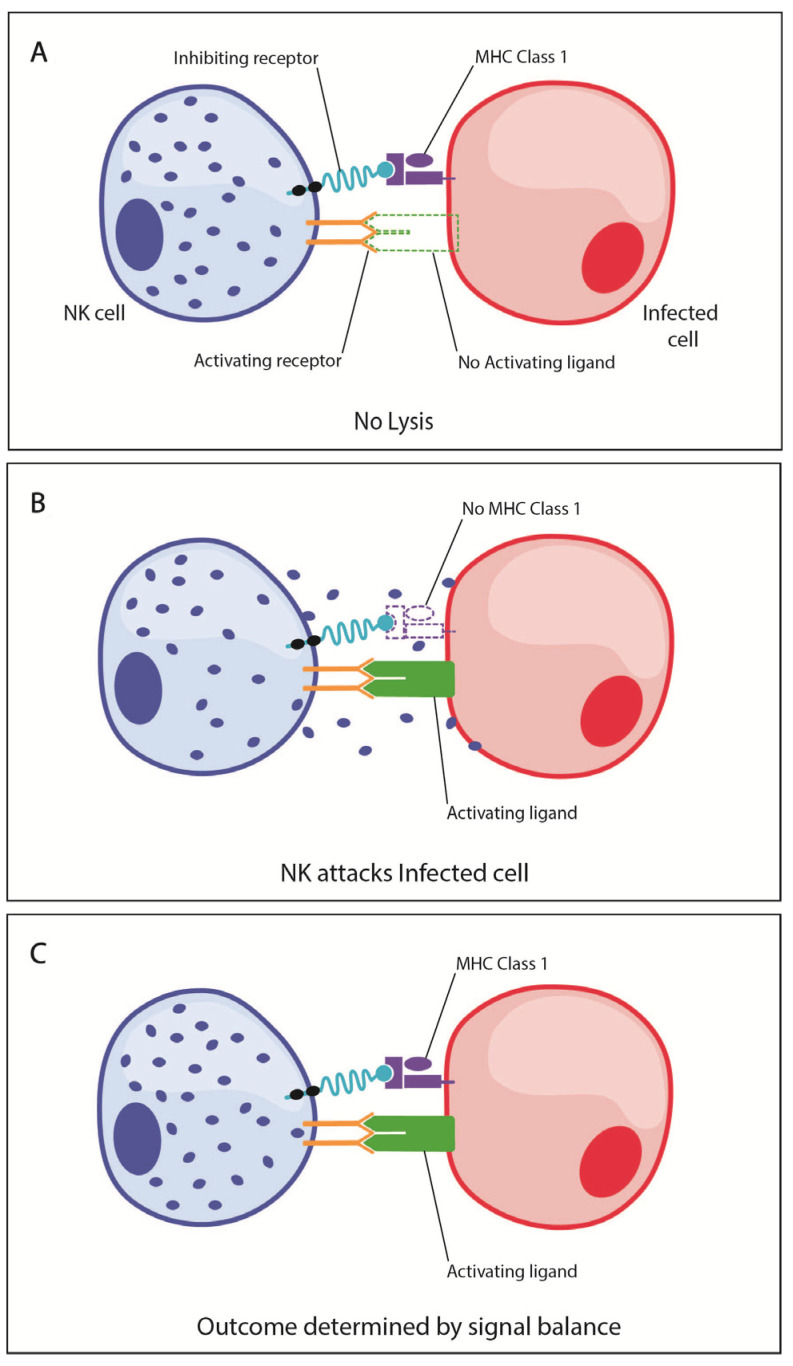
Natural killer (NK) cell activating and inhibitory signaling mediates phenotypic outcomes. (**A**) When NK cell inhibitory receptors bind MHC class I on the target cell, this interaction suppresses NK cell activation and lysis of the target cell. (**B**) In some circumstances, the activating receptor on NK cells will bind the activating ligand on the target cell, while the inhibitory receptor does not bind MHC Class I, leading to NK cell activation and lysis of the target cell. (**C**) When the target cell expresses both MHC Class I and activating ligands, the fate of the target cell is determined by a balance of signals.

When an NK cell interacts with a target, activating and inhibitory receptors on the NK cell engage cognate receptors on targets cells, and the combination of receptor engagement determines target cell outcomes. When MHC class I is present on the target cell, the NK cell activating and inhibitory receptors engage simultaneously, suppressing NK cell activation and lysis of the target cell (Figure 1A). In the absence of MHC class I, engagement of activating receptors in the absence of MHC class I-dependent inhibitory signals leads to activation of the NK cell and lysis of the target cell (Figure 1B). When the target cell presents both MHC class I and activating ligands, the fate of the target cell is determined by a balance of signals (Figure 1C). This balance is determined by the amount of activating and inhibitory receptors on the NK cells and also the number of ligands expressed by the aberrant cell [69].

NK cells develop from a variety of tissues, including bone marrow (BM), liver, and spleen. While not completely understood, immature NK cells depart the BM and undergo differentiation in secondary lymphoid organs, where the majority of NK cells have a CD56^bright^ phenotype, indicative of potential for further differentiation and/or activation [70]. These NK cells have undergone licensing or education, in the BM or other developmental tissues, whereby inhibitory receptors bind to self-MHC molecules to render them tolerant to self-MHC as well as enabling future recognition of the absence of self-MHC inhibiting receptors. The precise mechanisms are as yet unclear, but it is apparent that it results in variable expression of inhibitory receptors having functional consequences [70]. Furthermore, prior stimulation and engagement of NK cells with MHC class I molecules through their inhibitory receptors allows for a greater response when recognizing abnormal cells, this process is known as NK cell licensing (education) [71]. Although NK cell receptors do not undergo somatic rearrangement, studies suggest that the number and strength of inhibitory receptors engaged with MHC class I calibrates the potential response for cytotoxicity and cytokine secretion [72,73]. Phenotypic characterization of unlicensed NK cells from an MHC class I-deficient environment and mature MHC class I-stimulated NK cells revealed that mature NK cells express a greater number of activating and inhibitory receptors, and exhibit increased functional activity when stimulated with MHC class I [72,74]. The absence of NK receptors for self-MHC or the absence of MHC altogether results in NK cell hypo-responsiveness and tolerance to MHC-deficient cells [4,72]. However, NK cell licensing can be overcome during viral infection, as NK cells need to produce a robust response against virally-infected cells down-regulating MHC class I, for early elimination of pathogens and minimize disease progression [75].

## 3. NK Cell Phenotypic Subsets

**Figure 2:**
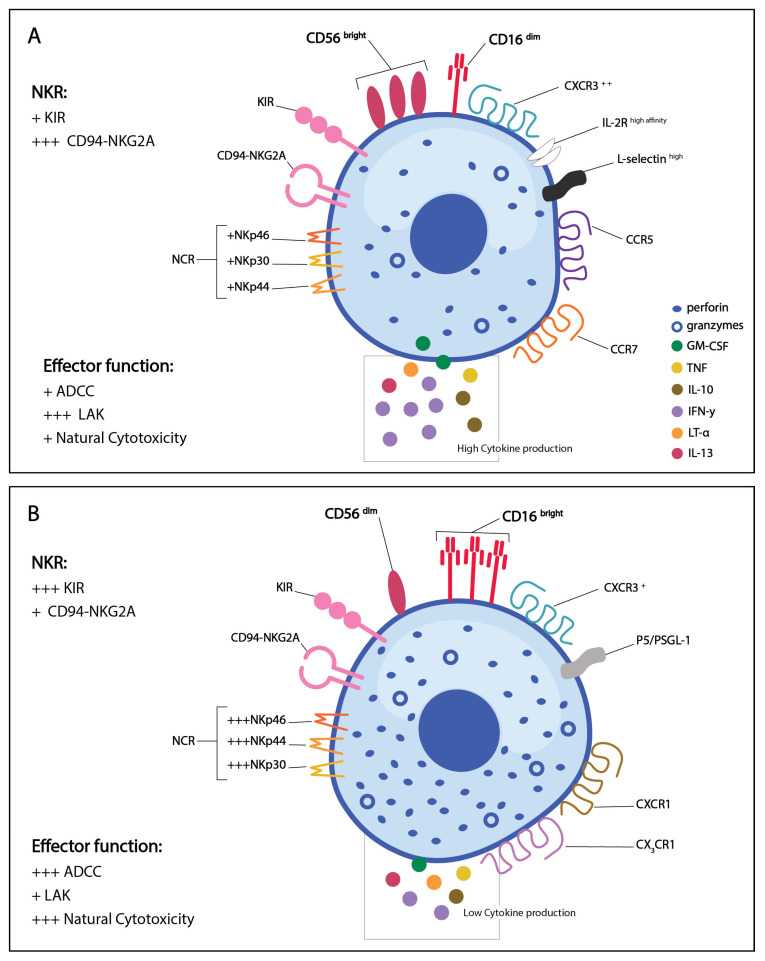
Phenotype of activated human NK cell populations. (**A**) CD56^bright^ CD16^dim^ NK cells are functionally higher in cytokine production and lymphokine-activated killer activity, and have low ADCC and natural cytotoxicity. (**B**) CD56^dim^ CD16^bright^ NK cells are functionally lower in cytokine production and lymphokine-activated killer cytotoxicity but have high ADCC activity and natural cytotoxicity. + = low expression/functionality, +++ = high expression/functionality. Figure adapted from Cooper et al. [24].

Two distinct phenotypic populations of circulating NK cells have been identified to date. These two populations are based on the cell surface density of CD56 and CD16 (Figure 2). CD56 is an isoform of the human neural-cell adhesion molecule expressed on the surface of human NK cells and the function of CD56 on NK cells in currently unclear [76,77]. In contrast, CD16 is a low affinity FcγRIII found on the cell surface of human NK cells, and acts on antibody coated targets to direct antibody-dependent cellular cytotoxicity (ADCC) [24]. Although CD56 is not significant for NK cell functionality, certain cell-surface markers have unique functions on CD56^dim^ and CD16^bright^ human NK cell subsets. CD56 expression levels have also been associated with NK cell activation, and rare populations of CD56^-^ NK cells have been associated with disease states [77]. NK cell ontogenesis was recently thoroughly reviewed [70,78], so we will only briefly discuss this topic here.

Approximately 15% of circulating lymphocytes in humans are NK cells [5,6,7]. The CD56^bright^ CD16^dim^ subset comprises about 10% of the NK cells in peripheral blood and spleen, but 75–90% of NK cells in LNs [70,79]. In contrast, the CD56^dim^ CD16^bright^ subset makes up the remaining 90% of circulating and splenic NK cells in humans [24,70,79]. Approximately 10–20% of lymphocytes in the lung are NK cells, with the majority being CD56^dim^ CD16^bright^ [26,70].

All resting NK cells, regardless of subset, express medium affinity IL-2 receptor for IL-2 mediated lymphokine-activated killer (LAK) cytolytic activity and proliferation whereas the high affinity IL-2 receptor is expressed on the CD56^bright^ population only [80,81] (Figure 2). Additionally, L-selectin is generally restricted to the CD56^bright^ population [82] whereas CD56^dim^ express PEN5/P-selectin glycoprotein ligand 1 (P5/PSGL-1) [83]. CD107a, a lysosomal membrane protein that is trafficked to the surface of NK cells upon degranulation can be used as a marker for NK cell activation [84,85].

CD56^bright^ CD16^dim^ NK cells are known for their capacity to produce a variety of cytokines following activation, specifically, granulocyte-macrophage colony-stimulating factor (GM-CSF), tumor necrosis factor (TNF), lymphotoxin-alpha (LT-α), interferon gamma (IFN-γ), interleukin (IL)-10 and IL-13 [24,26]. The low expression of CD16 limits Fc-mediated ADCC responses, hence the ability to lyse target cells is functionally low. The CD56^bright^ CD16^dim^ population has low expression of KIRs and NCRs and high expression of the inhibitory receptor CD94- NKG2A [24,83,86]. CD56^bright^ CD16^dim^ NK cells express the high-affinity IL-2 receptor (IL-2R), L-selectin, and multiple chemokine receptors including CCR5, CCR7, and high levels of CXCR3 for recruitment to the site of infection [82,87] (Figure 2A). CD56^bright^ CD16^dim^ NK cells are generally considered immunomodulatory, with the potential to drive pro- or anti-inflammatory immune responses through differential cytokine production. For example, IFN-γ and TNF-α production promote Th1 responses along with macrophage and dendritic cell activation during influenza virus infection. Alternatively, CD56^bright^ CD16^dim^ NK cells can produce IL-10 during chronic viral infections to regulate CD8 T cell responses and mitigate tissue damage [26]. CD56^bright^ NK cells are thought to be activated, produce cytokines, and differentiate into more mature CD56^dim^ NK cells [26,70].

The CD56^dim^ CD16^bright^ population express higher levels of the NCRs, are more granular, and have increased cytotoxic activity compared to CD56^bright^ CD16^dim^ NK cells [24,26]. The high expression of CD16 enables increased ADCC and a greater ability to lyse target cells. This population expresses the intermediate affinity IL-2R, has high expression of the KIRs, and low expression of inhibitory receptors such as the C-type lectin CD94-NKG2A [24,83,86]. CD56^dim^ CD16^bright^ NK cells lack L-selectin but express PEN5/P-selectin glycoprotein ligand 1 (P5/PSGL-1)) [83] (Figure 2B). The biology and function of CD56 NK cell subsets have been limited to in vitro studies using human NK cells as murine NK cells, which are analogous to human NK cells, do not express CD56 and comparable murine subsets have proven difficult to identify [76,88,89].

## 4. NK Cell Activating Receptors–the Natural Cytotoxicity Receptors

**Figure 3:**
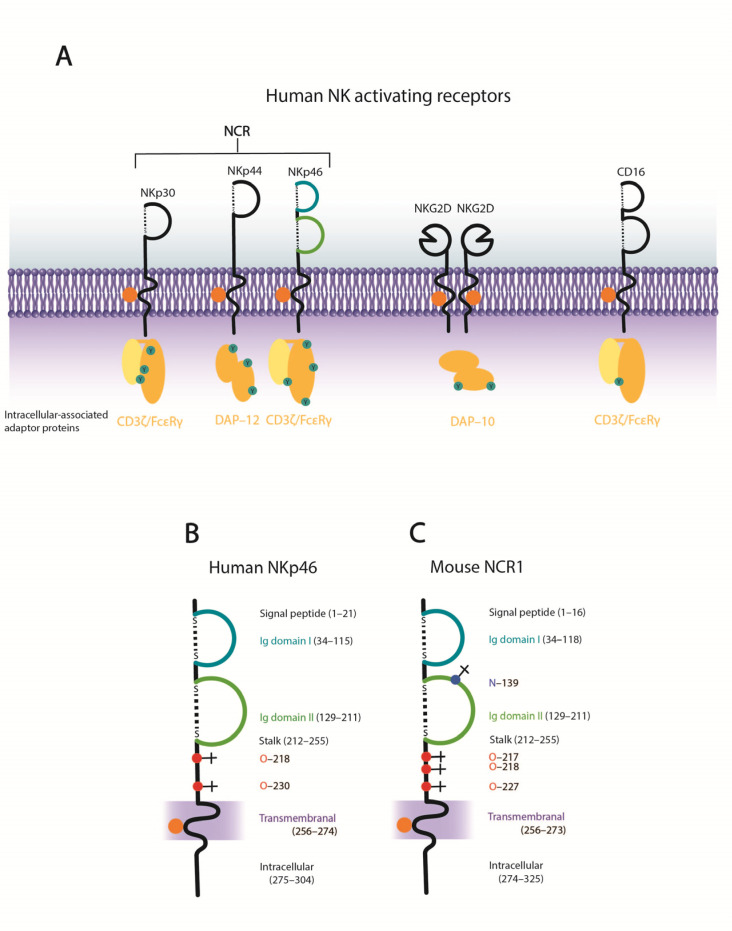
NK cell activating receptors. (**A**) Schematic of human NK cell activating receptors. (**B**) Structural features of hNKp46. In addition to the two predicted *O*-linked glycosites in the stalk, an additional two *O*-linked glycosites in the intracellular domain are predicted (NCBI: CAA04714). (**C**) Structural features of murine NCR1 (NCBI: NP_034876). Protein domain predictions were performed using SMART protein domain annotation resource [100]. *N*-linked glycosylation predicted performed using NetNGlyc 1.0 server [101], and *O*-linked glycosylation prediction performed using NetOGlyc 4.0 server [102]. Panel (**A**) modified from Arnon et al. [103], panels (**B**,**C**) modified from Glasner et al. [22].

**Figure 4:**
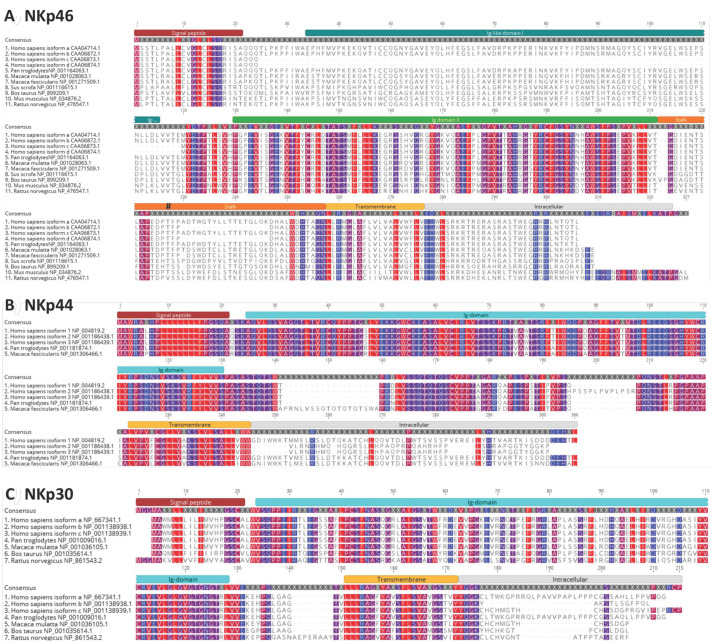
Amino acid sequence alignment of natural cytotoxicity receptor homologs from various species. (**A**) NKp46, (**B**) NKp44, and (**C**) NKp30. Protein domains are indicated above consensus sequence. Sequence alignments were performed using MUSCLE. Conserved amino acids are highlighted. Protein domain predictions were performed using SMART protein domain annotation resource [100]. Predicted *O*-linked glycosylation site (Thr225) identified to be crucial to HA and hNKp46-Ig interactions indicated by hash sign. NCBI protein accession numbers are indicated.

Major NK cell activating receptors include the NCRs, NKG2D, and CD16 (Figure 3A). Other activating receptors include NKG2C-CD94 [90] and the co-activating receptor 2B4 [91]. Human NCRs include NKp46, NKp44, and NKp30, are structurally conserved (Figure 4) and belong to the Ig-superfamily. NKp30 and NKp44 are comprised of one Ig-like domain [92,93], while NKp46 is comprised of two C2-type Ig-like domains [94]. NKp46 and NKp30 are constitutively expressed on resting and activated NK cells [54,92], whereas the expression of NKp44 is induced following IL-2 activation [95]. Murine NK cells do not express NKp44 or NKp30, however, *NKp30* is present as a pseudogene in inbred laboratory mice and a soluble form may be expressed in the Ryukyu mouse (*Mus carolis*) [96]. NCRs are highly expressed on certain populations of NK cells, namely, the CD56^dim^ CD16^bright^ population (Figure 2). Importantly, NCR surface expression levels have been shown to be positively correlated with NK cell cytolytic activity [97,98,99] and NK cell mediated cytolysis of normal and tumor cells can involve synergistic signaling through multiple NCRs, or lysis can be mediated through individual NCRs [92].

The signaling cascade for NK cell activation is similar for most activating receptors. Kinases are recruited through immunoreceptor tyrosine-based activating motifs (ITAMs) for downstream phosphorylation and subsequent activation of NK cells. All three NCRs associate with ITAM-containing molecules via a positively charged amino acid in the transmembrane domain that interacts with a negatively charged amino acid in the transmembrane domain of cognate signaling partner [12,104,105,106]. NKp46 and NKp30 associate with CD3ζ chain homodimers or with CD3ζ/FcRγ heterodimers [92,106], while NKp44 associates with homodimeric intracellular protein, DAP12 [107,108] (Figure 3). Inhibitory receptors recruit phosphatases through immunoreceptor tyrosine-based inhibitory motifs to inhibit downstream phosphorylation and activation of NK cells [108].

It has been suggested that engagement of a single NCR can lead to the activation of the other NCRs through cross-talking that leads to amplification of the signaling cascade [109]. In the context of pathogen infections, antibody blockade of both NKp46 and NKp30 results in a synergistic dampening of NK cell cytolytic activity against *Plasmodium falciparum*-parasitized erythrocytes [51]. It has been reported that HIV-1 infection functionally impairs NK cytolytic activity by way of downregulation of all NCRs [98], this may explain the increased tumor incidence in HIV^+^ patients.

### 4.1. NKp46

NKp46 was the first NCR identified and is named based on the 46 kDa band isolated from human NK cells [94]. Functionally, NKp46 engagement leads to NK cell mediated lysis of transformed cells [54,94,97]. Human NKp46 (hNKp46) is known to have a functional ortholog in mice, termed NCR1 [105]. To determine the cellular expression of NKp46, Gazit et al. generated an *NCR1*^gfp/gfp^ knockout mouse and found that NCR1 (NKp46) expression was restricted to NK cells [21]. All GFP^+^ cells also expressed DX5^+^ (a murine NK cell marker), but not CD3 or B220, markers for murine T and B cells [21]. However, it has been shown that NKp46 is expressed on certain T cell populations in mice and human [110,111,112]. NKp46 orthologs have been identified in non-human primates, swine, bovine, and rats [113,114,115,116] (Figure 4A), in addition to other species such as black-flying foxes, horses and seals. To date, NKp46 has not been identified in chickens [117] or ducks, which are important for the maintenance and transmission of (avian) influenza viruses.

NKp46 is considered to be a major NK cell lytic receptor and evidence suggests that these cells are activated against a variety of targets, however the target cell ligands are still largely unknown [103,118]. Surface expressed heparin sulfate proteoglycans have been reported as a NKp46 (and NKp30) ligand ligand [55]. Interestingly, NKp46 can bind to human pancreatic β cells and could lyse β cells via a NKp46-dependant mechanism [119]. More recently, complement factor P (properdin), a glycoprotein found in serum was identified as a ligand for NKp46. While complement factor P is not expressed on the surface of NK cell targets, it may be part of a larger multimeric protein complex found on the surface of target cells [120].

Many viral, bacterial, fungal, and parasitic pathogens activate NK cells via NKp46 (Table 1). Vimentin has been identified as the NKp46 ligand on *Mycobacterium tuberculosis* (TB)-infected monocytes [49]. Although evidence for NCR interactions between fungal ligands are scarce, NKp46 has been shown to interact with *Candida glabrata* Epa1, Epa6, and Epa7 adhesion molecules [62].

Interactions between NCRs and viral pathogens are supported by numerous studies. Of these, influenza A virus hemagglutinin andNKp46 interactions have been extensively studied and will be described in detail below.

### 4.2. NKp44

NKp44 is expressed on human and non-human primate NK cells [95,114] (Figure 4B) and orthologs have been identified in multiple other species such as pigs, horses, and black flying foxes. To date, NKp44 has not been identified in chickens or ducks. NKp44 is not normally expressed on human resting NK cells, although its expression is induced following IL2 activation [95]. NKp44 has also been reported to be expressed on plasmacytoid dendritic cells [121], and T cells [110,122]. The cellular ligand for NKp44 on neoplastic cells was identified in 2005. Termed NKp44L, it is a truncated isoform of mixed lineage leukemia-5 protein (also known as inactive histone-lysine N-methyltransferase 2E) (NCBI accession: NP_891847). NKp44L localizes to the cellular membrane as a result of its unique C-terminal sequence, where it is then able to interact with NKp44 [123]. Subsequently, proliferating cell nuclear antigen has also been identified as a cellular ligand of NKp44 [124].

U2 cells chronically infected with human immunodeficiency virus 1 (HIV-1) induce expression of NKp44L [56], which is likely to mediate increased recognition by NK cells for clearance of HIV infected cells. Expression of the cellular ligand was blocked by anti-gp41 polyclonal sera, suggesting that gp41 influences NKp44L expression, or may itself interact with NKp44. In contrast, Kaposi’s sarcoma-associated herpesvirus (KSHV) has been shown to downregulate NKp44L expression in vitro [44], a possible mechanism to evade NK cell recognition. NKp44L is pivotal for recognition of tumorigenic or transformed cells as it is expressed on aberrant cells and absent from normal tissues [123].

### 4.3. NKp30

To date, NKp30 expression has been identified on humans and non-human primate NK cells [92,125] (Figure 4C) and orthologs have been identified in other species such as pigs, horses, and black flying foxes. Similar to NKp44, NKp30 has not been identified in chickens or ducks. Along with NKp46, NKp30 is constitutively expressed on all resting and activated NK cells [92] and has also been identified on some T cell subsets [126,127,128]. Although *NKp30* is a pseudogene in most mouse strains, it is functional in the Ryukyu mouse, *Mus caroli* [96]. NKp30 can synergize with the other NCRs in lysing tumor-bearing cells [92]. NKp30 may also play an immunomodulatory role. Dendritic cells can activate NK cells through NKp30 signaling signaling [129], and NK and dendritic cell interactions though NKp30 signaling can modulate dendritic cell maturation [130], or lysis. DC lysis is particularly potent when NKp30 and NKp46 signaling synergizes [131].

Several cellular ligands have been identified for NKp30, including heparin sulfate proteoglycans [55], B7-H6 [132] and the nuclear human leukocyte antigen-B-associated transcript 3 [133]. NKp30 has been shown to interact with human cytomegalovirus (HCMV) pp65 protein, reducing cytolytic activity via CD3 zeta chain dissociation, potentially reducing NK cell lysis of immature dendritic cells, a reservoir for latent HCMV [42].

A growing body of evidence highlights NCRs contribution to defense against other pathogens, in addition to transformed cells, and this is currently still a subject of intense investigation. As described earlier, there has been extensive research investigating the interactions of NK cell NCRs with various pathogens. The commonality with most pathogens is that the protein interacting with NCR is a lectin. Of these pathogens, influenza virus has been shown to interact extensively with NCRs NKp46 and NKp44, but not NKp30 [21,22,28,29,30,39,58]. In particular, the surface glycoprotein of IAV, HA, which is a lectin and functions to bind sialylated glycan receptors on the surface of the host cell membrane, has been shown to interact with NK cell NCRs. To provide further insights into NCR and IAV HA interactions, it is necessary to understand NCR glycobiology and IAV HA functional biology.

## 5. Glycobiology of Human NK Cell NCRs

To characterize the sialylated glycobiology of human CD56^+^ CD3^-^ NK cells, Owen et al. stained human NK cells with lectins that are specific for either α2,3-linked sialic acids (*Maackia amurensis*), or α2,6-linked sialic acids (*Sambucus nigra*), and found that both α2,3- and α2,6-receptor linkages are present on human NK cells. Moreover, basal levels of α2,3-linked sialic acids on human NK cells was higher [57]. The presence of surface expressed sialylated glycans would facilitate the interaction of viral lectins with NK cells. The glycostatus of the NK cell NCRs has been characterized predominately by in silico methods and are described in detail below.

### 5.1. NKp46

Contemporary *in silico* prediction of hNKp46 occupied *O-* and *N-*linked glycosylation sites reveals that hNKp46 (NCBI: CAA04714 (isoform a)) is predicted to contain four occupied *O*-linked glycosites (serine (Ser) 218, threonine (Thr) Thr230, Ser288, Ser291) and no occupied *N*-linked glycosites [101,102]. Of the four predicted *O*-linked glycosites in hNKp46, only Ser218 and Thr230 are extracellular, and both potential glycosites are located on the stalk domain (Figure 3 and Figure 4). Contemporary algorithms predict NCR1 (NP_034876) to have one occupied *N*-linked glycosite (Asparagine (Asn) 139), and three *O*-linked glycosites occupied (Ser217, Ser218, Ser227) [101,102], whereas previously it was reported to possess *N*-linked glycosites only (Asn139, Asn216 and Asn238) [105]. As per hNKp46, all *O*-linked glycosites in NCR1 are located within the stalk domain, whereas the *N*-linked glycosite is located in the membrane proximal Ig domain II (Figure 3 and Figure 4).

When NKp46 (isoform a) was first cloned in 1998 (NCBI: nucleotide: AJ001383; protein: CAA04714), it was predicted to contain one *N*-linked and two *O*-linked glycosylation sites [94]. At this time, Thr125, Thr225, and Asn216 glycosites were predicted to be occupied. However, as described above, contemporary algorithms do not predict that these specific sites harbor glycans. Thr225 was initially predicted to be occupied (with research focused on this residue)-contemporary algorithms now predict Thr230, rather than Thr225, to be glycosylated. However, it is best to exercise caution when interpreting specific *O*-linked glycosites-rather than focusing on the specific amino acid, it is suggested that the local protein region is likely to harbor an *O*-linked modification [102]. Robust experimental analysis of glycosites is needed to confirm computationally predicted glycostatus of NKp46.

Initial biochemical analysis of hNKp46 by Sivori et al. [54] suggested that hNKp46 does not contain occupied *N*-linked glycosites (in concordance with contemporary computational predictions), although it does contain occupied *O*-linked glycosites. The absence of *N*-linked glycosites on hNKp46 was confirmed biochemically by Vitale et al. [95]. Subsequently Pessino et al. [94] reported conflicting results, suggesting that hNKp46 is *N*-linked glycosylated. However, this study combined PNGase F treatment, which is specific for *N*-linked oligosaccharides, with additional glycan hydrolyzing enzymes that target both *N*- and *O*-linked glycans, weakening the conclusions [94]. HPLC analysis of *O*-linked glycans present on COS-7-expressed hNKp46, revealed that NKp46 contains both α2-3- and α2,6-linked sialic acids, present on both linear and branched *O*-linked glycans [31].

### 5.2. NKp44

Computational prediction of occupied *O-* and *N-*linked glycosylation sites in hNKp44 (NCBI: NP_004819) reveals one *N*-linked glycosite (Asn180), and extensive *O*-linked glycosylation, with 18 potential glycosites predicted (Ser130, Ser133, Thr136, Thr138, Ser139, Thr141, Ser147-148, Thr150, Thr152, Ser154, Thr159, Ser168, Ser170, Thr171, Ser176, Ser181, and Thr182) [101,102]. The potential *N*-linked glycosite is located extracellularly, close to the transmembrane domain. All predicted *O*-linked glycosites are located extracellularly–Ser130 is specifically located within the Ig-domain (Figure 4).

When hNkp44 was first cloned in 1998, biochemical analysis of rhNKp44 suggested that it was indeed *N*-glycosylated [95], however, the authors concluded that hNKp44 did not harbor *O*-linked glycosites (although the biochemical data pertaining to *O*-linked deglycosylation was not shown). Subsequent computational analysis of hNKp44 by Cantoni et al. [93,134] suggested that hNKp44 (NCBI: CAB39168) contained one *N*-linked glycosite (Asn180), and 13 *O*-linked glycosites and were all predicted to occur extracellularly.

### 5.3. NKp30

In contrast to the extensively glycosylated hNKp44, and to some degree, hNKp46, hNKp30 is less extensively glycosylated. Initial computational characterization of hNKp30 (NCBI: CAB54004) identified two *N*-linked glycosites (Asn42 and Asn121) and no *O*-linked glycosites were identified [92]. Reanalysis of the glycostatus of hNKp30 (NCBI: CAB54004) using contemporary algorithms suggests the presence of only one *N*-linked glycosite (Asn121), and no *O*-linked glycosites [101,102]. Computational prediction of occupied *O-* and *N-*linked glycosylation sites in humanNKp30 (NCBI: NP_667341) reveals only one *N*-linked glycosite (Asn121) and one *O*-linked glycosite (Thr168) [101,102]. The predicted *N*-linked glycosite is located within the Ig-domain, whereas the predicted *O*-linked glycosite is located within the intracellular domain (Figure 4). Collectively, this information suggests that hNKp30 generally harbors one predicted *N*-linked glycosite, present in the Ig-domain, and no extracellular *O*-linked glycosites.

As described above, the exact number and type of sialyated glycans on the human and murine NCRs remains unclear. An in-depth analysis of NCR glycans is needed to clarify whether predicted glycosites are indeed occupied, and of biological relevance. MALDI mass spectrometry analysis of wild-type and mutated hNKp46, including the various isoforms, would provide robust insight as to the number of occupied glycosites, in addition to structure and branching of glycans present on NKp46. Although computational predictions of occupied NCR glycosites have merit, verification with experimental data is ultimately needed to clarify whether predicted NCR glycosites are occupied or not. This information would be useful in determining mechanisms that facilitate interactions with viral lectins.

## 6. Role of NK Cells in Influenza Infection

Influenza A viruses continue to threaten the human population by way of seasonal epidemics and sporadic pandemics. Seasonal epidemics of influenza A viruses cause approximately 3–5 million cases of severe illness and 290,000–560,000 deaths annually [135]. NK cells are recruited during the early phase of influenza infection, with the peak of NK cell activation and recruitment to the lung occurring ~5–6 days post influenza infection [136,137]. Recruitment of NK cells to the sites of infection is mediated by several chemokines including CCL3 (macrophage inflammatory protein 1α), CCL4 (macrophage inflammatory protein 1β), and CXCL10 (IFN-γ-inducible protein 10) [138]. Specifically, recruitment of NK cells to influenza infected lungs is dependent on CXCR3 chemokine receptor expression, and CCR5 chemokine receptor expression is required for localization of NK cells to influenza infected epithelial cells [136]. Following recruitment and activation, NK cells secrete a variety of cytokines and cytotoxic granules (Figure 2). Tissue resident NK cells initially described as unresponsive can be activated by influenza infection, but additional studies are needed to define stimulatory factors and functional potential or these resident NK cells [26,139,140]. Upon recruitment to the lung, both CD56^bright^ and CD56^dim^ NK cells have been shown to be activated and produce INF-γ, perforin and granzymes, although CD56^bright^ had greater proliferative marker expression while CD56^dim^ cells expressed greater perforin suggesting greater effector function [139,140]. However, whether these are resident or infiltrating NK cells is unclear [26]. IFN-γ production by NK cells enhances the cytotoxic activity of NK and infiltrating CD8^+^ T cells and may be contribute to resolution of influenza infection or pathology [26]. Additional studies are needed to clarify the impact of the NK cell cytokine response during influenza infection. Elimination of influenza infected cells by activated NK cells is mediated by the cytotoxic granules, perforin and granzymes. Perforin generates pores in the target cell membrane and granzymes triggers a signaling cascade that leads to apoptosis. Alternatively, death receptor-mediated cytotoxicity of influenza infected cells can be mediated by Fas ligand or TNF-related apoptosis inducing ligand mechanisms (reviewed in [141]).

Numerous studies investigating the role of NK cells in influenza pathogenesis suggested that NK cells are protective. Depletion of (Asialo GM-1^+^) NK cells was associated with increased morbidity and mortality in the murine model [142], and influenza infection of *NCR1* knockout mice was lethal [21]. However, other studies have reported contrasting results where depletion of Asialo GM-1^+^ or NK1.1^+^ NK cells was shown to be protective following influenza infection and was associated with decreased lung pathology [143,144]. In the murine model, NK cell depletion had no effect on lung virus titers [143,145] suggesting that NK cells have a pathological role in influenza infection. Further, NK cell depletion also impaired influenza-specific NP_366–374_ CD8^+^ T cell responses [145]. Most of these reports assessed outcomes in C57BL/6 mice using A/Puerto Rico/8/1934 (H1N1) as the challenge virus. However, this strain is extensively lab-adapted with different variants having dramatically different lethal doses. In fact, different stocks of the same virus can have distinct phenotypes in vivo, depending on the infectious particle to non-infectious (defective-interfering (DI)) particle ratio [146]. It is possible that the A/Puerto Rico/8/1934 variants may differentially activate or infect NK cells or differentially activate type I and/or type III interferons, altering the subsequent inflammatory environment in the lung. Specific studies using a defined influenza virus strains or A/Puerto Rico/8/1934 variants would address these possibilities.

Work from Zhou et al. may also explain some of the differences seen across the various studies. The impact of NK cell depletion on outcome of infection was assessed using a range of inoculum doses. Antibody depletion of NK1.1^+^ NK cells improved survival rate with a high-dose infection, while NK cell depletion resulted in reduced survival with a medium inoculum dose, and there was no difference in survival between control and NK cell-depleted mice infected with a low inoculum dose [147]. The data suggest that disease severity may alter the impact of NK cells on outcomes of infection. Whether virus virulence has a similar effect as virus dose remains to be tested. Finally, age of the mice during infection has also been suggested as a variable across studies with increasing frequencies of NK cells in the lung as mice age [144].

Influenza viruses predominately infect mucosal epithelial cells of the respiratory tract, however IAV can also infect human NK cells in vitro and induce NK cell apoptosis, which would counteract their function during the early immune response [148]. This result has been subsequently confirmed in vivo [149]. Importantly, the ability of different IAVs to activate NK cells varies. Highly pathogenic H5N1 avian influenza virus and 1918 pandemic influenza virus pseudotyped particles were able to more robustly activate NK cells than pdmH1N1 pseudotyped particles [32]. While the mechanisms of differential activation are unclear, the variable stimulation of NK cells may contribute to altered IAV infection outcomes in vivo.

## 7. Functional Biology of Influenza a Virus Hemagglutinin

**Figure 5:**
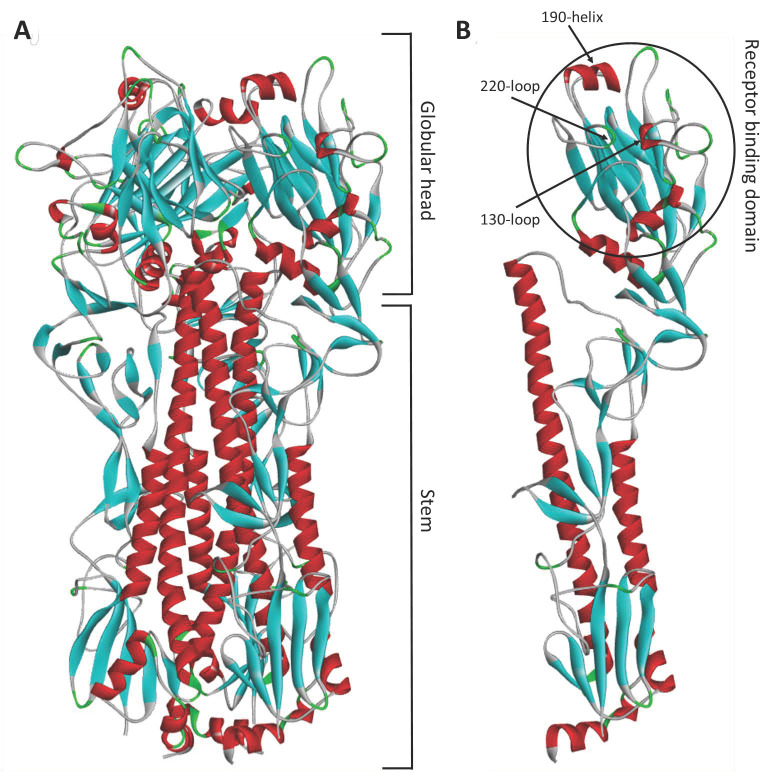
Protein homology model of influenza A virus haemagglutinin. Protein homology model of A/Puerto Rico/8/1934 (H1N1) (NCBI: ABO21709), based on the crystal structure of 1HA0 [156]. (**A**) Haemagglutinin trimer. (**B**) Haemagglutinin protomer. Protein homology model was generated with SWISS-MODEL [157,158,159], and visualized in Discovery Studio (Dassault Systemès BIOVIA).

Two influenza A surface glycoproteins, HA and neuraminidase (NA), have been shown to interact with NK cell NCRs, and these surface glycoproteins are critical to the life cycle of influenza A and B viruses. To understand mechanisms driving these interactions, it is necessary to understand the functional biology of these glycoproteins. Influenza (A and B) virus host cell binding and fusion is mediated by [150,151] and NA is crucial for progeny virus release from infected cells [152,153]. The HA glycoprotein is a lectin (carbohydrate binding protein), and is present on the surface of the virion as a trimer of protomers [154] (Figure 5A). Each HA protomer is composed of two structural domains, (1) the membrane-proximal stem domain, which upon cleavage by host cell serine proteases, liberates the fusogenic peptide, and (2) the membrane-distal globular head, which contains the receptor binding site (RBS) [155] (Figure 5B).

The IAV HA glycoprotein binds to sialic acid receptors on surface of the host cell membrane [160]. Upon HA binding to sialic acid receptors, the virion is internalized by receptor-mediated endocytosis and exposure to the low pH in the endosome induces an irreversible conformational change of the cleaved HA glycoprotein [161,162], resulting in the fusion domain being inserted into the host cell membrane and subsequent fusion of viral and host membranes [161,163,164]. Following virus replication, packaging, and virus assembly at the host cell membrane, the NA glycoprotein cleaves sialic acid receptors bound by newly formed virions, enabling the release of progeny virions from the infected cell.

As described above, NK cell NCR interactions with invading pathogens seem to be mediated by interactions with viral lectins, the primary function of which is to bind host cell receptors. The RBS of influenza A HA, located on the distal globular head domain, contains conserved secondary structure features that interact with sialic acid receptor moieties; namely, the 130-loop, the 220-loop, and the 190-helix [165] (Figure 5B). Host cell receptors are bound within the RBS by hydrophobic interactions, in addition to hydrogen bonding with the 130- and 220-loops, and conserved amino acids located at base of the RBS: Y98, W153, H183, and Y195 (H3 numbering used throughout) ) [151,165,166]. Site directed mutagenesis of these conserved amino acid residues (Y98F, H183F, and L194A) almost completely abolishes receptor binding [167].

The host range of influenza virus is determined by several factors, a major determinant being the expression pattern and configuration of sialic acid receptors on host tissues [168,169]. There are two major types of influenza A virus receptors, based on the linkage of the sialic acid moiety to the penultimate galactose within carbohydrate side chains [151] human influenza viruses have an almost exclusive preference for α2,6-linked sialic acids, whereas avian influenza viruses preferentially bind α2,3-linked sialic acids [170]. However, there are exceptions to this rule, human influenza viruses isolated early in the 20th century such as A/London/1/1918 (pdmH1N1) [171] and A/New York/1/1918 (pdmH1N1) [172] (although not all 1918 pandemic isolates possess dual receptor specificity), A/Puerto Rico/8/1934 (H1N1) [170,173,174], A/Fort Leonard Wood/1/1952 (H1N1) [174], and A/Roma/1/1949 (H1N1) [174] exhibit dual receptor specificity. The recent H7N9 influenza viruses also exhibit dual receptor specificity, mediated by residues G186V and Q226L on the H7 HA [175]. Structural analyses of influenza receptor specificity have revealed that human influenza viruses with α2,6 sialic acid specificity exhibit interactions beyond the RBD, with HA residues D190 and D225 contributing to favorable receptor interactions. In contrast, avian HA receptor binding (α2,3 sialic acid specific) is mediated primarily by the sialic acid moiety, with no detectable interactions beyond the RBD contributing to glycan interactions [173].

## 8. Influenza Haemagglutinin Interactions with NK Cell NCRS

The ability of the influenza haemagglutinin glycoprotein to interact with, and functionally activate, NK cells have been well documented since the 1980s. Initial studies reported the functional activation of NK cells by IAV HA glycoprotein [52,53]. These studies demonstrated that NK cell cytolytic activity was blocked by pre-treatment of virus with anti-HA antisera [53], or by pretreatment of NK cells with either whole virus, or purified HA glycoprotein [52].

### 8.1. Inlfuenza A Virus HA Interaction with NKp46

The first study describing the interaction of IAV and NK cell NKp46 was in 1998 by Mandelboim et al. [28]. In this maiden study, the authors cloned the extracellular domain of hNKp46 isoform b (NCBI: AJ006121) and fused it will the Fc domain of human IgG1. It is unclear whether the conserved glycosylation sites present in the IgG1 Fc domain [176,177] remained intact or were mutated. The hNKp46-Ig fusion protein recognized SeV- and IAV-infected cells, and this interaction was abrogated by anti-SeV hemagglutinin-neuraminidase (HN) or anti-IAV HA mAb blockade, respectively. NKp46-Ig binding to SeV HN-transfected cells recapitulated results using virally infected cells and suggests that the SeV HN glycoprotein is mediating the interaction with hNKp46-Ig. Additionally, Mandelboim et al. reported that hNKp46-Ig blocked IAV-mediated agglutination of sheep erythrocytes (data was not shown)-sheep erythrocytes almost exclusively express α2,3-linked sialic acids [178], suggesting that 2,3-linked sialic acids play a role in IAV and NKp46 interactions. Virally-infected cells (IAV or SeV) or SeV HN transfected cells also induced NK cell effector functions, which was abrogated by anti-NKp46 antisera, anti-HN (SeV) or anti-HA (IAV) antibody blockade. Pretreatment of NKp46-Ig with purified IAV HA abrogated binding to IAV-infected cells. Finally, inhibition of IAV NA activity enhanced binding of NKp46-Ig to IAV-infected cells and NA-treatment of NKp46-Ig reduced NKp46-Ig binding to IAV-infected cells–these results suggest that the interaction is sialic acid-dependent and is mediated by influenza virus HA.

### 8.2. Inlfuenza A Virus HA Interaction with NKp44 and NKp30

Building upon this initial study, Arnon et al. demonstrated that IAV and SeV infected cells, in addition to SeV HN transfected cells, interact with hNKp44-Ig, but not hNKp30-Ig [39]. This interaction was also abrogated by anti-HA (IAV) or anti-HN (SeV) antibody blockade, or pretreatment with purified HA (IAV). Anti-NA antibody blockade increased IAV interactions with NCRs [39], confirming results previously reported by Mandelboim et al. [28]. IAV-infected cells more readily engage with hNKp44-Ig than hNKp46-Ig [29]. This observation aligns with the predicted glycostatus of the NCRs–hNKp44 is more heavily glycosylated than hNKp46 and is likely to have increased binding opportunities simply due to number of glycosites present.

### 8.3. Identification of NCR Domain that Engages with Influenza A Virus HA

Human NKp46 isoforms a and b possess two Ig domains, Ig-like domain I and Ig domain II (domain II is not present in isoforms c or d) (Figure 4A). To elucidate the domain interacting with IAV HA and SeV HN glycoproteins, Arnon et al. [29] generated two truncated hNKp46-Ig fusion proteins: (1) hNKp46D1-Ig fusion protein encoding amino acids 1–100 (NCBI: AJ006121), and (2) the hNKp46D2-Ig truncated fusion protein encoded the 24 amino acid signal peptide of CD5, followed by residues 101–235 of hNKp46 (NCBI: AJ006121). Specifically, hNKp46D1-Ig encoded Ig domain I, whereas hNKp46D2-Ig encoded Ig-domain II and the stalk domains. hNKp46D2-Ig is predicted to contain *O*-linked glycosites, whereas hNKp46D1-Ig does not contain any predicted glycosites. Using these tools, the proximal Ig-like and stem domains of NKP46 were shown to interact with IAV HA [29]. It has also been shown that *Plasmodium falciparum* interacts with the proximal Ig-like domain of NKp46 [51]. NKp30 and NKp44 have three and six isoforms, respectively, however NKp44 splice variants have not been shown to have altered lectin binding [179]. While single nucleotide polymorphisms (SNPs) have been reported for all three NCRs [180], there is no data suggesting potential SNPs alter NCR interactions with HA or other pathogen glycoproteins.

### 8.4. Sialylated Glycans Are Required for Influenza A Virus HA to Interact with NCRs

*O*-linked glycosylation sites are present in the membrane proximal stem domain of hNKp46D2-Ig and desialylation abolished interactions with IAV or SeV infected cells further suggesting that the NKp46 interaction with pathogen lectins is a sialic acid-dependent event. To provide insights to the receptor linkage mediating the interaction (α2,6 vs. α2,3), truncated fusion proteins were produced in CHO cells, rather than COS-7 cells, resulting in the production of fusion proteins with α2,3-linked sialylation only (CHO cells lack functional α2,6-sialyltransferase [181]). This abolished the interaction of human influenza viruses with hNKp46-Ig. However, a major caveat is that all IAVs tested exhibit a strict preference for α2,6-linked sialic acids. Further, Mandelboim et al. had previously reported that hNKp46-Ig blocked binding of IAV (A/Puerto Rico/8/1934 (H1N1)) to sheep erythrocytes [28]. Sheep erythrocytes almost exclusively express α2,3-linked sialic acids [178], which suggests that α2,3-linked sialic acids do contribute to IAV HA and hNKp46-Ig interactions.

To determine the glycosite(s) that were mediating the interaction of IAV with hNKp46-Ig, Arnon et al. [29] mutated the three predicted hNKp46 glycosites: T125A, N216A, and T225A/N (current algorithms predict four *O*-linked and no *N*-linked glycosites). Mutation of T225/N *O*-linked glycosite diminished NKp46-Ig binding to IAV-infected cells (of note, the cell line used was LCL 721.221 cells (ATCC CRL-1855) and are not typically used in the influenza field, it would be of interest to repeat this using MDCK cells). Thr225 is located in the stalk domain, and is conserved in all species investigated, with the exception of rNKp46, which contains valine at this position [113,182] (Figure 4). Mutation of Thr125 or Asn216 generally had minimal effect on binding, although Asn216 exhibited some strain dependent variation–this aligns with the contemporary in silico predictions that these particular sites are likely not glycosylated. Additional studies by Achdout et al. [58] confirm that Thr225 is likely involved, Thr125 has little influence on binding, and that Asn216 may play a role.

In a follow up study characterizing IAV HA interactions with murine NCRs, Glasner et al. [22] reported that *N*-linked glycosylation is crucial for HA and (mouse) NCR1-Ig interactions. NA or PNGase F treatment of NCR1-Ig abolished interactions with IAV ‘coated’ cells, and *O*-glycanase cocktail treatment had little effect. NA treatment of NCR1-Ig reduced binding to influenza (A/Puerto Rico/8/1934 (H1N1)) infected MDCK cells, although this reduction was minimal. An N139A NCR1-Ig mutant demonstrated marginally reduced binding to MDCK-infected cells, however a triple mutant (N139A N216A N238) did not reduce binding. Collectively, Glasner et al. concluded that although *N*-linked glycosylation was crucial for IAV HA interaction with NCR1, the three *N*-linked glycosites in NCR1-Ig (N139, N216, N238) were not crucial and that other *N*-linked glycosites must contribute.

In a follow up study, Glasner investigated the role of *O*-linked glycosylation on IAV HA and NCR1-Ig binding [35]. Confirming previous results, a triple *N*-glycosylation NCR1-Ig mutant did not reduce binding, although in this study they concluded that *N*-linked glycosylation was not a requirement. Following mutagenesis of the predicted *O*-linked residues Thr222 and Thr225, reduction in binding was observed. As per hNKp46, NCR1 Thr225 was identified to be critical to IAV and NCR1 interactions [35], highlighting this sialylated *O*-linked glycosite as an important contributor to IAV HA and NCR1 interactions.

### 8.5. Neuraminidase Treatment of NCRs Abrogates HA Binding

Several studies have demonstrated that sialidase/NA treatment of the NKp46/NKp44, to remove terminal sialic acid moieties, reduces binding [23,28,29,34,36,40,46,61], highlighting that the sialic acid is predominant factor facilitating interactions. In contrast, inhibition of NA enzymatic activity by the anti-NA drug, oseltamivir carboxylate, results in increased recognition of NKp46-Ig by IAV HA [23,34,36] and NDV HN [40].

### 8.6. Sialic Acid Linkage and NCR Recognition

However, despite providing evidence that the interaction is indeed a sialic acid-dependent event, characterization of IAV HA with α2,6- vs. α2,3 binding preferences to NK cell NCRs remains understudied. Numerous studies have described the interaction of NK cell NCRs with human pandemic influenza viruses, human seasonal influenza viruses, and swine influenza viruses. Some studies have investigated avian influenza virus interaction with NCRs, although there remains a paucity of data (Table 1). The influenza A virus that has been most extensively studied is the human-origin, highly lab-adapted isolate, A/Puerto Rico/8/1934 (H1N1) (Table 1). This virus has been shown to exhibit dual receptor specificity in some instances [173] and two different stocks of this virus has even been reported to exhibit opposite binding preferences [183]. Hence, the use of A/Puerto Rico/8/1934 (H1N1) may complicate conclusions. Rather than selecting isolates according to seasonal or pandemic classification, selection of viruses based on their receptor specificity will provide further insight into mechanisms driving NCR and IAV HA interactions.

One such question pertains to interactions of avian influenza viruses with the NCRs. Avian influenza viruses are classified as one of two pathotypes, based on their pathogenicity characteristics in chickens. Highly pathogenic avian influenza viruses (HPAIVs) cause severe disease and high mortality rates and low pathogenicity avian influenza viruses cause no to mild disease and low mortality rates [184,185,186]. Despite the stark contrast in disease manifestation, the receptor binding preferences of both pathotypes are the same. Thus, binding analyses utilizing both pathotypes should lead to the same binding outcomes. Several studies have investigated avian influenza virus interactions with the NCRs. Two studies utilized the low pathogenicity A/VNH5N1-PR8/CDC-rg vaccine strain (modified to represent a low pathogenicity virus with a monobasic cleavage site motif) [30,58], one used H5N1 HPAIV virus-like particles (VLPs) [32], and another used cells transfected with H5N1 HPAIV HA [30]. H5N1 HPAIV HA was shown to interact with hNKp44, and elicit NK cell degranulation and IFN-γ production [30]. Induction of NK cell degranulation and IFN-γ production by H5N1 HPAIV was subsequently confirmed when HPAIV VLPs were used [32]. However, this NK cell activation was not recapitulated when hNKp46 was investigated [58]. It was demonstrated that hNKp46-Ig interacted with both A/Puerto Rico/8/1934 (H1N1) and A/VNH5N1-PR8/CDC-rg-infected cells (suggesting a role for α2,3-linked sialic acids), although, the A/VNH5N1-PR8/CDC-rg vaccine strain did not induce NK cell cytolytic activity. While both HPAIV and low pathogenicity isolates were capable of interacting with NCRs, the functional activation outcomes of NK cells differed. Thus, there is conflicting data on the impact of HA ligand specificity on NCR ligation and NK cell activation.

## 9. Concluding Remarks

Experimental approaches used to elucidate IAV HA and NCR interactions include staining of virally-infected cells with the NCR fusions proteins, staining of HA transfected cells with the NCR fusion proteins, ELISA using recombinant proteins, and antibody blockade of HA and NCR interactions. However, there is a need to delineate whether binding is mediated by a pathogen protein, or a pathogen-induced cellular factor [40]. Supporting evidence for NCR interactions with viral glycoproteins on IAV-, SeV-, or NDV-infected cells arise from anti-HA or anti-HN antibody blockade. The evidence for other pathogens is not so clear. For example, it has been reported that NK cells NCRs interact with TB [48,50], VACV [45], HSV1 [43], malaria [51], HMPV [41], and WNV [47], and that this interaction is abrogated following an anti-NCR antibody blockade. hNKp46 was found interact with a ligand present on HMPV-infected cells, however binding studies of human and mouse NCR1 revealed that the interaction was not mediated by HMNV proteins [41]. Furthermore, TB-infected monocytes were demonstrated to interact with NKp46-Ig, however it was determined that NKp46-Ig was interacting with a cellular ligand (vimentin), rather than a pathogen derived ligand [49]. Inclusion of pull-down experiments, in addition to mass spectrometry, as employed by Arnon et al. [42] and Garg et al. [49] will directly address whether NCR interactions are with a pathogen-derived protein, or a cellular factor that has been induced as a result of infection.

Whilst there has been considerable progress in elucidating the mechanisms of IAV HA and NCR interactions, some questions remain unanswered. The incredible complexity of potential glycan receptors calls for a robust analysis of the glycan structures and sialic acids present on the natural cytotoxicity receptors. An in-depth glycan analysis by mass spectrometry and/or liquid chromatography may provide insight as to why HA is able to bind NKp46 and NKp44, though not NKp30 nor CD99. Is this due to variation in glycan architecture present on each of these proteins? This example highlights the need to clarify the glycobiology of the NCRs, which would shed light on the intricacies of IAV and NCR interactions, and that further analyses into glycan structures mediating this interaction are necessary. Robust binding assays, such as solid-phase binding assays or biolayer interferometry, will serve to confirm protein:protein or protein:carbohydrate interactions (and their affinity), and eliminate any uncertainty posed by assays utilizing virally-infected or ‘coated’ cells.

Moreover, inclusion of avian and human IAVs with defined sialic acid binding properties would help to clarify whether IAV HA interactions with NCRs are sialic acid linkage specific. Comparisons of IAV and NK cell NCR interactions using a panel of influenza viruses with defined binding specificities (α-2,3 and α-2,6), hosts (avian, swine and human) and pathotypes (HPAIV and LPAIV) would be of immense interest. Importantly, inclusion of reverse genetics generated influenza virus receptor binding mutants (such as Y98F, H183F, and L194A mutants, described above), in robust binding assays will provide compelling evidence that interactions are mediated by HA binding to sialic acid receptors. Finally, a crystal structure of HA in complex with NKp46 would confirm the nature of IAV HA and NK cell NCR interactions. Ultimately, cross-disciplinary research encompassing immunology, virology, structural biology, and biochemistry will help to clarify mechanisms of influenza a virus HA and NK cell NCR interactions.

## Figures and Tables

**Table 1:** Selected literature describing viral, bacterial, and parasite interactions with the natural killer cell natural cytotoxicity receptors.

Pathogen (Species)	Isolate	NCRs Investigated	Summary	Reference
*Influenza A virus*	A/Bangkok/1/1979 (H3N2)	-	Pre-treatment of effector cells with purified HA blocked NK cell cytolytic activity. Suggests HA binds and functionally activated NK cells.	Ali et al. 1984 *Immunology* [52]
	A/Victoria/1/1975 H3N2)			
*Influenza B virus*	B/Lyon/1979			
*Influenza A virus*	A//Port Chalmers/1/1973 (H3N2)	-	Whole IAV or rHA functionally activates NK cells. Pre-treatment of IAV with anti-HA antisera or anti-HA F(ab’)_2_ blocked NK cell cytolytic activity.	Arora et al. 1984 *J. Virol.* [53]
*-*	-	hNKp46, hCD16	Biochemical analysis of hNKp46. Suggests that hNKp46 does not harbor *N*-linked glycosites, though does possess *O*-linked glycosites.	Sivori et al. 1997 *J. Exp. Med.* [54]
*Influenza A virus*	A/Puerto Rico/8/1934 (H1N1)	hNKp46, hCD16	SeV-infected cells bind NKp46-Ig. Cells transfected with SeV rHN bind NKp46-Ig. IAV HA binds NKp46-Ig, but not CD16-Ig or CD99-Ig. NA-treatment of hNKp46-Ig reduced binding by IAV. Lysis of SeV or IAV infected cells is reduced following anti-HN or anti-HA mAb treatment, respectively.	Mandelboim et al. 2001 *Nature* [28]
*Murine respirovirus*	Sendai virus			
*Influenza A virus*	A/Puerto Rico/8/1934 (H1N1)	hNKp44, hNKp30, hNKp46, hCD16	Recognition of transformed cell lines by NKp46-Ig, NKp44-Ig, NKp30-Ig, and CD16-Ig, but not CD99-Ig. NKp44-Ig bound SeV or IAV infected, or SeV HN transfected cells.	Arnon et al. 2001 *Eur. J. Immunol.* [39]
*Murine respirovirus*	Sendai virus			
*Mycobacterium tuberculosis*	H37Ra strain (avirulent) H37Rv strain (virulent)	hNKp46	NKp46 blockade with antisera reduced NKp46-mediated NK cell lysis of infected monocytes.	Vankayalapati et al. 2002 *J. Immunol.* [48]
*Murine respirovirus*	Sendai virus	hNKp46	hNKp46-Ig, but not hCD99-Ig, binds to SeV infected cells. Blockade of HN and hNKp46 binding with anti-HN mAb reduced binding.	Achdout et al. 2003 *J. Immunol.* [38]
*Influenza A virus*	A/Beijing/262/1995-like (H1N1)	hNKp46, hNKp44, hNKp30	SeV, IAV, and IBV bind NKp44-Ig and NKp46-Ig, but poorly to NKp30-Ig, and not at all to CD99-Ig. NA-treatment of NKp46-Ig reduced binding by SeV or IAV. Interaction with NKp46 specifically occurs with the proximal C2-type Ig-like domain. Suggests α-2,6 sialic acids on Thr225 critical for interaction.	Arnon et al. 2004 *Blood* [29]
	A/Moscow/10/1999-like (H3N2)			
	A/Sydney/5/1997-like (H3N2)			
	A/X-31 (A/Aichi/2/1968*A/Puerto Rico/8/1934) (H3N2)			
	A/X-127 (A/Beijing/262/1995* A/Puerto Rico/8/1934) (H1N1)			
	A/New Caledonia/20/1999 (H1N1)			
*Influenza B virus*	B/Yamanashi/166/1998 (Yamagata lineage)			
*Murine respirovirus*	Sendai virus			
-	-	hNKp46, hNKp30	Identified 6-*O*-sulfo-*N*-acetylglucosamine and hNK46 and hNKp30 interactions. Pretreatment of NKp30-Ig or NKp46-Ig with 6-*O*-sulfo-LacNAc reduced NCR binding to tumor cells. Pre-treatment with 3-*O*-sulfo-LacNAc or 4,6-di-*O*-sulfo-LacNAc did not affect binding. Additionally, heparin or heparan sulfate pre-treatment inhibited binding of NCRs to tumor cells.	Bloushtain et al. 2004 *J. Immunol.* [55]
*Human betaherpesvirus 5*	Human cytomegalovirus (AD169 strain)	hNKp46, hNKp44, hNKp30, hCD16	HCMV-infected cells bind NKp30-Ig, no binding to NKp46-Ig, NKp44-Ig, CD16-Ig or CD99-Ig was detected. Pulldown experiments identified HCMV pp65 protein bound to NKp30-Ig. Recombinant pp65 bound NKp30-Ig only (*K*_D_ 10 nM). Preincubation of NK cells with rpp65 blocked anti-NKp30 mAb binding. Counterintuitively, pp65 engagement of NKp30-Ig resulted in reduced NK cell cytolytic activity, resulting from CD3ζ chain dissociation from NKp30 complex.	Arnon et al. 2005 *Nat. Immunol.* [42]
*Human immunodeficiency virus 1*	Human immunodeficiency virus 1 (Sf2 strain)	hNKp44	HIV-1 infected cells induce NKp44L expression, and are highly susceptible to NK cell-mediated lysis. Ex vivo, NKp46L expressed on CD4^+^, but not CD8^+^, HIV positive individuals. NKp46L induced by gp41 (and its precursor, gp160). Anti-gp41 treatment of infected cells reduced NKp44L expression and blocked NK cytolytic activity.	Vieillard et al. 2005 *PNAS* [56]
*Influenza A virus*	A/Puerto Rico/8/1934 (H1N1)	msNKp46	IAV HA binds msNKp46-Ig. Generated *NCR1*^gfp/gfp^ knockout mice. Lack of NCR1 resulted in 100% mortality following IAV infection.	Gazit et al. 2006 *Nat. Immunol*. [21]
*Vaccinia virus*	Modified vaccinia virus Ankara	hNKp46, hNKp44, hNKp30	Vaccinia virus-infected cells recognize NKp30-Ig, NKp44-Ig, and NKp46-Ig. NKp30-Ig ligand was the most potently induced on virally-infected cells. Anti-NCR mAb blockade abrogated virally-infected cell:NCR interactions. Virally-induced ligand not directly identified.	Chisholm et al. 2006 *J. Virol.* [45]
	Vaccinia virus Western Reserve			
	Vaccinia virus Copenhagen			
	Vaccinia virus Wyeth			
	Vaccinia virus Lister			
	Vaccinia virus IHD-J			
	Vaccinia virus IHD-W			
	Vaccinia virus Tian-Tan			
	Vaccinia virus Tashkent			
	Vaccinia virus USSR			
	Vaccinia virus Patwadangar			
	Vaccinia virus King Institute			
	Vaccinia virus Dairen			
	Buffalopox virus			
	Rabbitpox virus (strain unknown)			
	Vaccinia virus Evans			
*Cowpox virus*	Cowpox virus Brighton Red			
	Elephantpox virus			
*Camelpox virus*	Camelpox virus (strain unknown)			
*Mycobacterium tuberculosis*	H37Ra strain (avirulent)	hNKp46	NKp46-Ig binds to TB-infected monocytes. Immunoprecipitation and mass spectrometry analysis of NKp46-Ig membrane bound ligands from TB-infected monocytes identified vimentin (type III intermediate filament) as a ligand of NKp46. Vimentin was shown to be surface exposed on TB-infected monocytes. Vimentin was also upregulated on Listeria-infected monocytes. Vimentin antisera reduced NK cell-mediated lysis.	Garg et al. 2006 *J. Immunol.* [49]
*Listeria monocytogenese*	Lm68 strain, Serovar 1/2b			
*Human alphaherpesvirus 1*	Herpes simplex virus 1 (strain 17) Herpes simplex virus 1 (strain F)	hNKp46, hNKp44, hNKp30	HSV1 virally-infected cells and HSV1 infected cell protein 0 (ICP0)-transfected cells are more susceptible to NK cell lysis. Cells infected with ∆ICP0 HSV mutant were less susceptible to lysis. Antibody blockade of the NCRs reduced NK cell lysis of HSV1-infected cells. Blockade of all three NCRs abrogate NK cell lysis of target cells. ICP0 is not surface expressed, thus ligand is likely to be cellular.	Chisholm et al. 2007 *J. Infect. Dis.* [43]
*Plasmodium falciparum*	FCR-3, D10, FP8, and 3D7 strains	hNKp46, hNKp30, hCD99	*P. falciparum* erythrocyte membrane protein-1 duffy binding-like 1α domain peptides bound NKp30-Ig, and minimally with NKp46-Ig. NKp46 and NKp30 bound to *P. falciparum* infected erythrocytes. NCRs bound to proximal Ig-like domain. Treatment with trypsin abrogated erythrocyte:NCR interaction. Blockade with anti-NKp46 or NKp30 reduced NK cell cytolytic activity.	Mavoungou et al. 2007 *J. Infect. Dis.* [51]
*Influenza A virus*	A/England/878/1969 (H3N2)	-	CD56^+^ CD3^-^ NK cells express both α-2,3 and α-2,6 sialic acids. Historical H3N2 virally-infected cells are lysed more efficiently by NK cells than those infected with contemporary H3N2 viruses. Treatment of NK cells with NA reduced NK-mediated lysis of IAV-infected cells.	Owen et al. 2007 *J. Virol.* [57]
	A/England/401/1985 (H3N2)			
	A/England/327/1990 (H3N2)			
	A/England/289/1993 (H3N2)			
	A/England/41/1996 (H3N2)			
	A/England/356/1996 (H3N2)			
	A/England/26/1999 (H3N2)			
	A/England/919/1999 (H3N2)			
	A/England/24/2000 (H3N2)			
*Mycobacterium avium*	NBL112/87 strain	hNKp46, hNKp44, hNKp30	*M. bovis* induced expression of NKp44 on CD56^bright^ NK cells, but not NKp30 or NKp46. All mycobacterium tested bound to NKp46-Ig. Additionally, *N. farcinica* (Gram-positive) and *P. aeruginosa* (Gram-negative) interacted with NKp44-Ig, minimally with NKp46-Ig, and not at all with NKp30-Ig. Electron microscopy revealed NKp44-Ig bound to surface of *M. bovis* and *E. faecium*, but not NKp46-Ig or NKp30-Ig. Interestingly, anti-NCR mAbs did not reduce *M.* bovis-induced NK cell activation, however, NKp44-Ig mAb reduced binding of NKp44-Ig to *M. bovis*.	Esin et al. 2008 *J. Virol.* [50]
*Mycobacterium smegmatis*	mc2 155 strain			
*Mycobacterium tuberculosis*	H37Rv strain (virulent)			
*Mycobacterium bovis*				
*Salmonella enterica*	Bacillus Calmette-Guérin (Pasteur) strain			
*Escherichia coli*				
*Streptococcus pyogenes*	Serovar Enteritidis			
*Enterococcus faecium*				
*Pseudomonas aeruginosa*				
*Actinomyces meyeri*				
*Cellulomonas denverensis*	DSM 15764			
*Nocardia farcinica*	DSM 43665			
*Influenza A virus*	A/VNH5N1-PR8/CDC-rg (A/Vietnam/1203/2004*A/Puerto Rico/8/1934) (H5N1–LPAIV) A/Cambodia/408008/2005 (H5N1–HPAIV) A/Puerto Rico/8/1934 (H1N1)	hNKp44, hNKp30	Infection of cells with IAV enhances NK cell mediated lysis. Cells transfected with IAV HA (H5N1–HPAIV) bind NKp44-Ig, poorly to NKp30-Ig. rHA binds hNKp-44-Ig (ELISA). IAV pseudoparticles bind to hNKp44 transduced cells.	Ho et al. 2008 *J. Virol.* [30]
*West Nile virus* *Dengue virus*	West Nile virus ISR98-Goo1 Dengue virus 1 FGA/89 Dengue virus 4 Burma 1976	hNKp44, hNKp46, hNKp30	Recombinant DENV and WNV E glycoproteins bind hNKp44-Ig. WNV rE glycoprotein bound membrane-associated hNKp44-Ig. Binding was also demonstrated for WNV VLPs (E and prM glycoproteins) and virally-infected cells. VLP and hNKP44-Ig interaction was augmented by low pH treatment. Interactions mapped to domain III of WNV E glycoprotein. Anti-NKp44 serum reduced WNV E binding to hNKp44-Ig and reduce NK cell cytolytic activity.	Hershkovitz et al. 2009 *J. Immunol* [47]
*Avian orthoavulavirus 1*	Newcastle disease virus (Ulster 2C strain) Newcastle disease virus (MTH-68/H strain)	hNK46, hNKp44, hNKp30	NDV-infected cells bind hNKp46-Ig and hNKp44-Ig, but not hNKp30-Ig. Treatment with anti-HN mAb reduced HN binding to hNKp46-Ig and hNKp44-Ig. HN or F transfected cells revealed interaction directly mediated by HN. Desialylation of hNKp46-Ig and hNKp44-Ig abrogated interaction with HN. NK cells lysis blocked when NDV cells treated with NA-inhibitor or anti-HN mAb.	Jarahian et al. 2009 *J. Virol.* [40]
*Influenza A virus*	A/Puerto Rico/8/1934 (H1N1)	hNKp46	Suggests Thr225 does not harbor a unique glycoform (compared to Thr125). Characterized α2,3- and α2,6- *O*-linked glycans present on HEK293T, CHO and COS-7 expressed hNKp46.	Mendelson et al. 2010 *J. Virol.* [31]
	A/Brisbane/59/2007 (H1N1)			
	A/New Caledonia/20/1999 (H1N1)			
*Influenza A virus*	A/VNH5N1-PR8/CDC-rg (A/Vietnam/1203/2004*A/Puerto Rico/8/1934) (H5N1–LPAIV) A/Puerto Rico/8/1934 (H1N1) A/Texas/1/1977 (H3N2) A/Swine/Israel/2009 (pdmH1N1)	hNKp46, hNKp30	Avian and human influenza viruses interact with hNKp46, although H5N1 HA:hNkp46 interaction did not induce NK cell-mediated lysis of H5 infected cells, whereas H1N1 did. Authors conclude no increase in lysis of H5 cells as H5 avian viruses have not evolved/adapted to humans.	Achdout et al. 2010 *J. Virol.* [58]
*Influenza A virus*	A/Brevig Mission/1/1918 (pdmH1N1) A/California/07/2009 (pdmH1N1) A/Anhui/1/2005 (H5N1–HPAIV) A/Brisbane/10/2007 (H3N2)	hNKp46	In contrast to Achdout et al. 2010, Du et al. H5N1 and 1918 pdmH1N1 HA (pseudoviruses) induced greater NK cell activation and lysis, than 2009 pdmH1N1 IAV. hNKp46 was found to be downregulated upon stimulation. Treatment with anti-NKp46 mAb lead to reduced CD69 expression.	Du et al. 2010 *J. Virol.* [32]
*Influenza A virus*	A/Hong Kong/54/1998 (H1N1)	-	Pre-treatment of NK cells with whole IAV virions or rHA inhibits NK cell cytolytic activity by way of downregulation of the CD3 ζ chain and cytolytic granule exocytosis (NKp46 and NKp30 surface expression levels remained unchanged). NA-treatment of human NK cells reduces rHA internalization.	Mao et al. 2010 *J. Virol.* [59]
	A/New Caledonia/20/1999 (H1N1)			
-	-	hNKp46	Glycan-binding analysis of *E. coli* expressed NKp46-His and sulfate- or neuraminic acid containing multimeric glycans. Recombinant hNKp46 bound heparin-BSA and heparan-sulfate-BSA in the low µM range; 2-*O*-linked, 6-*O*-linked, and *N*-linked sulfates important for interaction. Binding to Sialyl Lewis X-expressing transferrin also demonstrated.	Ito et al. 2011 *Biochem. Biophys. Res. Commun.* [60]
*Vaccinia virus* *Ectromelia virus*	Vaccinia virus (Western Reserve strain) Ectromelia virus (MP-Nü strain)	hNKp46, hNKp30, hNKp44	VV and ECTV infected cells upregulated ligands for hNKp46-Ig and hNKp30-Ig, but not hNKp44-Ig. ∆HA virus did not induce NCR ligands. Anti-HA mAb blocked HA hNKp46-Ig and hNKp30-Ig interactions. Desialyation/deglycosylation of hNKp46-Ig reduced binding with HA, though increased binding with hNKp30-Ig. Demonstrated that VV HA reduces B7-H6 binding to hNKp30. VV-infected cells less susceptible to NK cell-mediated lysis.	Jarahian et al. 2011 *PLoS Path.* [46]
*Fusobacterium nucleatum* *Influenza A virus*	PK1594 strain	hNKp46, msNKp46, hNKp44, hNKp30, hCD16	*F. nucleatum* bacterium bind NKp46-Ig and NCR1-Ig, minimally with NKp44-Ig, and not at all with NKp30-Ig, CD16-Ig. Interaction was not sialic acid-dependent; and was heat, proteinase K, and pronase sensitive.	Chaushu et al. 2012 *PLoS Path.* [33]
	A/Puerto Rico/8/1934 (H1N1)			
*Influenza A virus*	A/Puerto Rico/8/1934 (H1N1)	msNKp46	Binding of IAV HA to NCR1 is mediated by *N*-linked glycosylation. Deglycosylation of msNKp46 with PNGase F abolished binding. Deglycosylation of msNKp46 with *O*-linked glycanase cocktail had little effect on binding. Surprisingly, mutation of Asn139, Asn216 or Asn238 *N*-linked glycosites did not affect binding.	Glasner et al. 2012 *PLoS ONE* [22]
-	-	hNKp44, hNKp30	Glycan-binding analysis of *E. coli* expressed NKp44-His and NKp30-His to sulfate- or neuraminic acid containing multimeric glycans. Recombinant hNKp46 and hNKp30 bound heparin-BSA in the low to mid nM range. NKp44, but not NKp30, bound Sialyl Lewis X-expressing transferrin. NA-treatment of transferrin abrogated binding.	Ito et al. 2012 *Biol. Pharm. Bull*. [61]
*Human gammaherpesvirus 8*	Kaposi’s sarcoma-associated herpesvirus	hNKp44	NKp44-Ig recognizes KSHV-infected cells. KSHV ORF54 downregulates NKp44L surface expression on KSHV-infected cells.	Madrid & Ganem 2012 *J. Virol.* [44]
*Influenza A virus*	A/Puerto Rico/8/1934 (H1N1)	hNKp46, msNKp46,	Mortality and lung virus load of IAV-infected *NCR1*^gfp/gfp^ knockout mice is dose-dependent. NCR1 is not protective at high IAV challenge doses. IAV NA impairs sialic acid-dependent HA recognition of NKp46-Ig. Inhibition of NA activity augments HA binding of NKp46-Ig. Blockade of NA augments NK cell cytolytic activity. NA treatment of IAV-infected *NCR1*^gfp/gfp^ mice reduces mortality.	Bar-On et al. 2013 *Cell Rep.* [23]
	A/Texas/1/1977 (H3N2)			
*Influenza A virus*	A/Puerto Rico/8/1934 (H1N1)	hNKp46, msNKp46, hNKp44, hNKp30, KIR2DS4, KIR2DL1	NA hinders NCR interactions with IAV HA. Inhibition of IAV NA activity increases NK cytolytic function.	Bar-On et al. 2014 *J. Infect.Dis.* [34]
*Influenza A virus*	A/Puerto Rico/8/1934 (H1N1)	msNKp46	Analysis of NCR1 glycosylation status. Identified occupied *O*-linked glycosites at Thr222 and Thr225. T225A and T225A mutation reduced lectin binding. Reported that *O*-linked glycosites are crucial for interactions with IAV HA and subsequent NK cytolytic activity.	Glasner et al. 2015 *Cell Disc.* [35]
*Candida glabrata*	BG2	hNKp46 NCR1	*Candida glabrata* adhesins Epa1, Epa6, and Epa7 (all of which are lectins) engage with hNKp46 and NCR1. Fungal clearance was impaired in NCR knockout mice.	Vitenshtein et al. 2016 *Cell Host & Microbe* [62]
*Influenza A virus*	A/Puerto Rico/8/1934 (H1N1)	hNKp46	mAb blockade of hNKp46 inhibits NKp46 mediated NK cytolytic activity. Inhibition of NA activity increased HA binding to NKp46-Ig. HA binding to NA-treated NKp46-Ig was reduced. Demonstrate that HA also binds the 2B4 and NTB-A SLAM-family receptors in a sialic-acid dependent manner.	Duev-Cohen et al. 2016 *Oncotarget* [36]
	A/Brisbane/59/2007 (H1N1)			
*Human metapneumovirus*	Human metapneumovirus (strain not reported)	hNKp46, msNKp46	HMPV-infection induces expression of NKp46-Ig/NCR1-Ig ligand(s) in vitro. Blockade of HMPV and NKp46-Ig/anti-NCR1 interaction with anti-NKp46/anti-NCR1 mAb reduced NKp46-mediated NK cytolytic activity. Despite this, HMPV proteins do not interact directly with NKp46-Ig/NCR*1*-Ig. NCR1 controls HMPV virus load in vivo.	Diab et al. 2017 *Eur. J. Immunol.* [41]
*Human respirovirus 3* *Influenza A virus*	Human pararainfluenza virus 3 (strain C-243) A/Puerto Rico/8/1934 (H1N1)	hNKp46, hNKp44	HPIV3 HN induces NKp44 surface expression on CD14+ monocytes more potently than IAV HA. The opposite holds true for NKp46 expression. This effect can be abrogated by anti-HN or anti-HA antibody treatment.	McQuaid et al. 2018 [37]

## Data Availability

No new data were created or analyzed in this study. Data sharing is not applicable to this article.
